# The burden of chronic diseases and cost-of-care in subjects with HIV infection in a Health District of Northern Italy over a 12-year period compared to that of the general population

**DOI:** 10.1186/s12889-016-3804-4

**Published:** 2016-11-09

**Authors:** Eugenia Quiros-Roldan, Michele Magoni, Elena Raffetti, Francesco Donato, Carmelo Scarcella, Giuseppe Paraninfo, Francesco Castelli

**Affiliations:** 1 University Department of Infectious and Tropical Diseases, University of Brescia and Brescia Spedali Civili General Hospital, Brescia, Piazzale Spedali Civili 1, 25123 Brescia, Italy; 2 Local Health Agency of the Brescia Province, Viale Duca degli Abruzzi 15, 25124 Brescia, Italy; 3 Unit of Hygiene, Epidemiology and Public Health, Department of Medical and Surgical Specialties, Radiological Sciences and Public Health, University of Brescia, Viale Europa 11, 25123 Brescia, Italy

**Keywords:** Burden of HIV, Cost for HIV care, Prevalence, Chronic diseases

## Abstract

**Background:**

The increase in life expectancy of HIV-infected patients has driven increased costs due to life-long HIV treatment and concurrent age-related comorbidities. This population-based study aimed to investigate the burden of chronic diseases and health costs for HIV^+^ subjects compared to the general population living in Brescia Local health Agency (LHA) over a 12-year period.

**Methods:**

LHA database recorded diagnoses, deaths, drug prescriptions and health resource utilization for all residents during 2003–2014. We estimated HIV prevalence and incidence, HIV-related mortality as well as prevalence of chronic diseases in HIV^+^ subjects. Observed/expected ratio of chronic diseases was calculated by indirect standardization with the general population as reference. Direct cost of HIV care and determinants were estimates across the period.

**Results:**

HIV prevalence increased from 220 to 307 per 100 000 person-years while incidence decreased from 16.1 to 10.8 per 100 000 person-years from 2003 to 2014. Prevalence of most comorbidities increased over time but it reduced significantly (annual mean change − 0.7 %) when adjusting for age and gender. Observed to expected ratio for each chronic disease in HIV^+^ subjects decreased over time. Cost of HIV^+^ cures increased (+25 %) mainly due to cost for drugs (+50 %) but it stabilized in recent years. CD4^+^ cell count at the time of diagnosis was an important predictor of cost for HIV management.

**Conclusions:**

Expenditures for HIV-infection are driven mainly by drugs cost and they have increased overtime. However, our findings suggest that spending on public health for HIV care can improve prognosis of HIV-infected patients, reduce transmission of HIV infection and reduce the global burden of chronic diseases, leading to a reduction of HIV global cost in the medium-long time.

**Electronic supplementary material:**

The online version of this article (doi:10.1186/s12889-016-3804-4) contains supplementary material, which is available to authorized users.

## Background

During the past twenty years, antiretroviral therapy (ART) introduction has dramatically modified the natural evolution of HIV infection, with decreased morbidity and increased survival of infected patients [1–4]. Due to the important decrease in mortality and consequent aging of HIV-infected people, HIV infection is responsible for an increasing burden on healthcare services. Treating with ART has resulted in a continuous increase of the total treatment cost, mainly because of the appearance of new, more effective but more expensive drugs during last years [5, 6]. The increase in life expectancy of HIV-infected patients [7, 8] has also driven increased costs due to life-long HIV treatment and concurrent age-related comorbidities [9].

In our previous population-based study performed in a province of North Italy with high HIV prevalence, HIV infection ranked third among chronic diseases in order of total cost per patient to the National Health Service, with a mean cost of 9 894€ in 2007 (ranging from 8 104€ for HIV–infected patients without comorbidities to 12 013€ for HIV infection plus cancer) [10]. However, an update of the estimates of cost-of-care of HIV^+^ subjects is needed because of recent changes of HIV epidemiologic pattern and guidelines of treatment, which have moved toward treating HIV^+^ patients earlier in the course of disease and have added newer, more effective and tolerated but also more expensive drugs [11].

The aim of this population-based study was to investigate the burden of chronic diseases and the direct health costs and resource allocation for HIV^+^ subjects compared to the general population living in the Brescia Local Health Authority (LHA) over a 12-year period, from 2003 to 2014.

## Methods

### Setting

This study was conducted in the Brescia Province, located in Lombardy Region (northern Italy) in the period 2003–2014.

The Spedali Civili General Hospital in Brescia is one of the largest public hospitals in Italy, and is also the reference hospital for the local School of Medicine, University of Brescia (University Hospital). It includes the only tertiary, referral center of infectious diseases and HIV of the area. In Italy, the National Health Service (NHS) provides universal coverage and is structured on three organizational levels: the central (the Ministry of Health), regional and local levels (Local Health Agency. LHA). In Italy, hospital service providers are paid on a fee-for-service basis, which is directly related to a system of Diagnosis-Related Groups (DRGs) [12]. Primary care and other out-patient services are based on a co-payment system for drugs, laboratory tests and any services provided to patients affected by chronic diseases. However, these services are fee-exempted for HIV^+^ subjects.

The study protocol was approved by the provincial Ethical Review Board. Written informed consent was obtained by all the patients enrolled.

### Population

We included in the analysis all residents in Brescia LHA registered in the Regional Health Service from 2003 to 2014. Non-documented migrants and non-EU citizens were excluded, as their access to care and utilization of health resources cannot be properly assessed as they can only access to emergency treatment under semi-anonymous condition.

HIV infection was identified using the following two sources, as previously described [10]:

- Brescia LHA electronic database. HIV diagnosis was based on HIV-related opportunistic illness requiring hospital admission (ICD9 = 042 or V08 diagnoses), receiving HIV drugs prescription or HIV reimbursement code assigned by the general practitioner or a specialist.
- Clinical charts and electronic databases of the Department of Infectious and Tropical Diseases, University of Brescia and Brescia Spedali Civili General Hospital. HIV diagnosis was based on positive HIV-1 or HIV-2-antibody test or positive HIV RNA.

For each year, we analyzed: a) HIV^+^ prevalent cases including subjects with HIV diagnosis who were present in the Brescia LHA database, independently of the year of HIV diagnosis; and b) HIV^+^ incident cases including subjects with HIV diagnosis in that year.

### The Brescia local health agency database

The Brescia Local Health Agency database (BLHADB) is a comprehensive and integrated information system including several databases tracking and listing all the health services provided by the NHS for each individual of the resident population [10].

Prescription of specific drugs is monitored for each individual by the BLHADB using the Anatomic and Therapeutic Chemical Classification (ATC) [13]. Each individual’s consumption of drugs is converted into a total number of daily defined doses (DDDs), according to the World Health Organization Collaborating Centre for Drug Statistics and Methodology [13]. Drug consumption data presented in DDDs provide a rough estimate of consumption and not an exact picture of each patient’s actual use. DDDs provide a fixed unit of measurement independent of price and formulation that enable to assess trends in drug consumption and to perform comparisons between population groups.

In this analysis we identified 10 families of chronic diseases using a set of ICD9-CM codes (see Additional file 1: Table S1). Each subject was considered to have a chronic disease if he had had Access to Continuity of Care (ACC) for one or more chronic diseases of interest collected in the BLHADB, according to at least one of the following criteria:

- receiving in-hospital or out-patient services with an ICD9-CM code for a chronic disease;
- receiving drugs for chronic diseases above a predefined DDD threshold (e.g. >10 % DDD of insulin or >30 % DDD of oral anti-diabetic drugs to define diabetes);
- having a chronic disease reimbursement code assigned by the general practitioner or a specialist;
- being admitted to a residential care or psychiatric facility.

ACC for chronic diseases has therefore been considered a proxy for disease prevalence.

### Outcomes and covariates

Information on vital status and date and cause of death were obtained from a record-linkage with the LHA Mortality Registry, based on each individual’s national registration code.

For each year of follow-up, we retrieved the presence of chronic diseases from BLHADB. The chronic diseases of interest evaluated were the following: cardiovascular diseases, liver diseases, dyslipidemia, diabetes, cancer, esophagus gastric duodenum diseases, neuropathies, severe psychiatric disorders, bronco-pneumopathy and kidney failure.

HIV infected persons who were at any time on antiretroviral treatment during the year were classified as ‘on antiretroviral treatment’ according to either hospital or LHA drug databases.

We evaluated the direct costs for each HIV^+^ subject, calculated as the sum of inpatient hospital costs and out-patients costs. Inpatient hospital costs (for both in-patient and day-care admissions) were based on DRG system as previously described [10]. Costs of out-patient consultations and examinations (laboratory and clinical imaging) were calculated based on the official standard costs assigned by the Italian Ministry of Health. All costs were annualized and expressed in nominal terms for the year in which they were incurred. Indirect Costs were not evaluated.

For patients followed by University Hospital we also retrieved CD4^+^ cell count at HIV diagnosis.

### Statistical analysis

HIV incidence and prevalence rates were calculated using incident and prevalent cases as numerators, respectively, and population living in the Brescia LHA during the period as denominator. Annual mean change was calculated using multivariate Poisson regression adjusted for age and gender.

We calculated the prevalence of chronic diseases in 3 years periods across the follow-up. To evaluate the temporal trend of risk of having a chronic disease, we performed an age-and gender-adjusted Poisson regression model for each chronic disease considered in this study. Results were expressed in terms of percentage of annual mean change of risk ([risk ratio-1]*100).

Prevalence of, and mortality for chronic diseases in HIV^+^ subjects were compared with those observed in the LHA general population using indirect standardization. Standardized prevalence ratios (SPRs) and standardized mortality ratios (SMRs) were computed adjusting for age and gender. The SPRs and SMRs and their 95 % confidence intervals (95 % CIs) were calculated using the Byar’s approximation of Poisson model. The SPRs were calculated for each chronic disease and for at least one, for each 3 year calendar period. The SMRs were also calculated for specific causes of death (HIV/AIDS, liver disease, suicide and cancer).

Association of chronic diseases, age, gender, period of follow-up and CD4^+^ cell count at baseline with per capita cost was evaluated by using multivariate regression models with per capita cost as the dependent variable with a bootstrap technique (1000 replications).

All statistical tests were two-sided, assumed a level of significance of 0.05 and were performed using Stata 12 software (Stata Statistics/Data Analysis 12.0-Stata Corporation, College Station, TX, USA).

## Results

### Characteristics of the HIV-infected population in the BLHA DB

From 2003 to 2014, a total of 4 621 subjects were identified as receiving care for HIV infection and included in this study. The main characteristics of the HIV-infected population are shown in Table 1. The number of HIV^+^ subjects increased from 2 302 in 2003 to 3 594 in 2014 with an increase in prevalence from 220 per 100 000 person-years in 2003 to 307 per 100 000 in 2014 (annual mean change +2.5 %, *p* < 0.001). We observed a decrease of HIV incidence rate from 16.1 to 10.8 per 100 000 person-years, (annual mean change − 4.5 %, *p* < 0.001), and of the overall mortality rate from 25.6 to 13.4 per 1000 HIV^+^ subjects, (annual mean change − 8.7 %, *p* < 0.001), with 542 deaths in the period (71 deaths for liver disease, 70 for cancer and 19 for suicide).

**Table 1:** Demographic and epidemiological characteristic of HIV^+^ subjects living in the Brescia Local Health Authority (LHA)

	2003	2004	2005	2006	2007	2008	2009	2010	2011	2012	2013	2014
Resident population (*n*)	1045478	1055256	1070896	1093708	1111659	1136817	1149520	1157391	1164382	1168168	1170158	1170655
Non-Italian citizens (%)	6.8	7.4	8.4	10.0	10.6	12.4	13.4	13.9	14.2	14.4	14.4	14.2
All patients receiving care for HIV infection												
Prevalence cases (*n*)	2302	2482	2651	2736	2935	2993	3181	3277	3369	3439	3527	3594
Prevalence rate per 100 000 person-years	220	235	248	250	264	263	277	283	289	294	301	307
Mean age (years)	40.1	40.7	41.3	42.1	42.8	43.7	44.4	45.1	45.8	46.4	47.2	47.8
Female (%)	28.6	28.4	29.2	29.6	29.5	29.3	29.9	30.1	29.9	29.9	29.7	29.5
Non-Italian citizens (%)	7.0	8.2	9.3	10.1	10.7	10.8	12.0	12.5	13.4	13.9	14.0	13.4
On antiretroviral therapy treatment (%)	67.3	70.7	70.1	73.9	75.5	76.8	78.1	79.3	80.2	82.0	82.0	82.5
New cases (*n*)	168	167	188	117	153	125	132	122	131	111	133	126
Incidence rate per 100 000 person-years	16.1	15.8	17.6	10.7	13.8	11.0	11.5	10.5	11.3	9.5	11.4	10.8
Mean age (years)	39.2	38.5	38.4	38.7	40.9	42.2	41.1	40.3	38.7	39.2	41.2	38.8
Female (%)	30.4	25.7	29.3	29.9	28.1	20.0	31.1	27.9	27.5	23.4	24.8	21.4
Non-Italian citizens (%)	18.5	16.8	19.7	24.8	20.9	19.2	31.1	23.8	31.3	27.9	29.3	23.8
Mortality (*n*)	59	46	46	37	48	47	48	46	46	43	28	48
Mortality rate per 1000 HIV^+^subjects	25.6	18.5	17.4	13.5	16.4	15.7	15.1	14.0	13.7	12.5	7.9	13.4

Among all HIV^+^ cases, the mean age increased from 40.1 years in 2003 to 47.8 years in 2014, so as prevalence of non-Italian citizens (from 7.0 % to 13.4 %) and of subjects on ART treatment (from 67.3 % to 82.5 %) (*p* < 0.001 for each variable). Considering only the new HIV^+^ cases, the mean age was stable around 40 years, but the proportion of females decreased over time (from 30.4 % to 21.4 %).

The distribution of CD4^+^ cell count at baseline in new HIV^+^ cases is shown in Table 2. About half of the patients (54 %) with newly diagnosed HIV infection had a late diagnosis (CD4^+^ cell count < 350 mm^3^) in the whole period without substantial change over time (*p* > 0.1).

**Table 2:** CD4^+^ cell count distribution of the new cases* of HIV infection at HIV diagnosis by 3 years-time periods

	2003–2005 n (%)	2006–2008 n (%)	2009–2011 n (%)	2012–2014 n (%)	Total
New cases	598	422	403	316	1739
CD4^+^ cell count mm^3^					
< 200	196 (32.8)	131 (31.0)	136 (33.7)	112 (35.4)	575 (33.1)
200–349	113 (18.9)	98 (23.2)	96 (23.8)	62 (19.6)	369 (21.2)
350–499	116 (19.4)	75 (17.8)	68 (16.9)	62 (19.6)	321 (18.5)
≥ 500	165 (27.6)	116 (27.5)	94 (23.3)	77 (24.4)	452 (26.0)
missing	8 (1.3)	2 (0.5)	9 (2.2)	3 (0.9)	22 (1.3)

*new cases were categorized in different periods according to the first access to hospital care

### Prevalence of chronic diseases

The prevalence of chronic diseases in HIV^+^ subjects according to observation period is shown in Table 3. The prevalence of at least one chronic disease decreased from 33.9 % in 2003 to 29.8 % in 2014 (annual mean change after adjustment for age and gender − 0.7 %; *p* < 0.001). Considering chronic diseases separately, we observed statistically significant trends, adjusting for age and gender of increasing of prevalence of dyslipidemia (annual mean change +5.5), diabetes (+1.6 %), and gastrointestinal diseases (+5.7 %) and decreasing prevalence of liver diseases (−4.8 %) and chronic respiratory diseases (−5.6 %). The crude prevalence of cardio-cerebrovascular diseases increased over time from 13.6 % in 2003 to 20.7 % in 2014 but this trend was not significant when adjusting for age and sex.

**Table 3:** Prevalence of chronic diseases in HIV population according to 3-year-time periods

Chronic diseases		2003–2005	2006–2008	2009–2011	2012–2014	Trend	Annual mean change^a^	*p*-value
Subjects observed in the period (n)		2846	3211	3604	3855			
Cardio-Cerebrovascular diseases	(*n*)	387	511	614	798	≈	+0.1 %	NS
	Prevalence (%)	13.6	15.9	17.0	20.7			
Liver diseases	(*n*)	573	640	547	428	↓	−4.8 %	<0.001
	Prevalence (%)	20.1	19.9	15.2	11.1			
Dyslipidemia	(*n*)	107	198	263	340	↑↑	+5.5 %	<0.001
	Prevalence (%)	3.8	6.2	7.3	8.8			
Diabetes	(*n*)	100	149	198	248	↑	+1.6 %	0.042
	Prevalence (%)	3.5	4.6	5.5	6.4			
Cancer	(*n*)	158	182	232	242	≈	+0.1 %	NS
	Prevalence (%)	5.6	5.7	6.4	6.3			
Gastrointestinal diseases	(*n*)	75	95	130	206	↑↑	+5.7 %	<0.001
	Prevalence (%)	2.6	3.0	3.6	5.3			
Neuropathies	(*n*)	110	136	146	166	≈	+0.2 %	NS
	Prevalence (%)	3.9	4.2	4.1	4.3			
Severe psychiatric disorders	(*n*)	96	118	141	129	≈	+1.1 %	NS
	Prevalence (%)	3.4	3.7	3.9	3.3			
Chronic respiratory diseases	(*n*)	129	135	122	154	↓↓	−5.6 %	<0.001
	Prevalence (%)	4.5	4.2	3.4	4.0			
Kidney failure	(*n*)	27	29	41	54	≈	+2.2 %	NS
	Prevalence (%)	0.9	0.9	1.1	1.4			
At least one chronic disease	(*n*)	966	1096	1150	1148	↓	−0.7 %	0.006
	Prevalence (%)	33.9	34.1	31.9	29.8			

^a^Annual mean change was calculated using multivariate Poisson regression models adjusted for age and gender with in offset the logarithm of general population. *NS p* > 0.05

### Comparison with the Brescia LHA general population

**Fig. 1:**
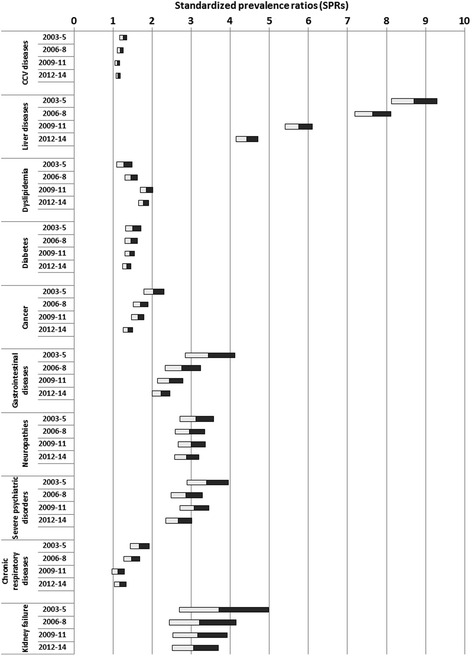
Standardized prevalence ratios (SPRs) and their 95 % confidence intervals (95 % CIs) of chronic diseases using Local Health Authority general population as reference. The filled segments of the horizontal bars represent the upper limit of the 95 % CI of the SPRs, whereas the open segments represent the lower limit

Figure 1 shows SPRs for chronic diseases using Brescia LHA population as the reference. The prevalence of any chronic disease was higher in HIV^+^ subjects than the general population, with total SPRs ranging from 1.15 to 6.18. However, SPRs decreased for all chronic disease except for dyslipidemia over time.

HIV^+^ subjects had a higher mortality than the LHA general population in the period, showing SMR of 5.94 (95 % CI 6.47–7.03). The SMR for all causes showed a steady decrease from 9.77 (95 % CI 11.53–13.53) in 2003–2005 to 3.43 (95 % CI 4.15–4.96) in 2012–2014. The mortality rates for liver disease, suicide and cancer were higher in HIV^+^ subjects than the LHA general population with SMRs of 5.65 (95 % CI 11.08–13.97), 3.55 (95 %, CI 5.90–9.21) and 1.60 (95 %, CI 2.06–2.60), respectively.

### Resource utilization and costs

The total health care cost for HIV^+^ subjects has increased substantially, from a mean of 28.5 million€ per year in 2007 to 36.5 million€ per year in 2014 (+22 %). The overall cost for public health care of subjects with chronic diseases in the Brescia LHA general population in 2014 are shown in Additional file 1: Table S2. HIV infection ranked as the third most costly chronic disease in the Brescia LHA in 2014.

Per capita health care costs by 3 years-time periods are shown in Table 4. Per capita costs increased from 8 271€ in 2003–05 to 10 945€ in 2009–11 (+32 %) with a slight decrease in 2012–14 (+ 25 % from the first to the last interval). The most important item of expenditure was ART, that increased from 5 079€ (61.4 % of the total per capita cost) in 2003–2005 to 7 562€ (72.9 %) in 2012–2014 (+ 49 %). Also in new HIV^+^ cases, the ART cost increased over time from 2 522€ (31.6 % of the total per capita cost) in 2003–2005 to 3 496€ (35.9 %) in 2012–2014 (+39 %). Of interest, in-patient costs decreased substantially (−28 %) in HIV^+^ subjects on ART, over the study period. However, in new HIV^+^ cases the most costly item of expenditure was in-hospital cost that was stable over time.

**Table 4:** Per capita cost in HIV^+^ subjects according to 3-year-time periods

	2003–2005	2006–2008	2009–2011	2012–2014
All HIV+ subjects (n)	2 846	3 211	3 604	3 855
Mean age (years)	40.7	42.9	45.1	47.2
*Per capita cost* (€)				
Total	8 271	9 870	10 945	10 374
In-hospital	1 648	1 497	1 386	1 183
Antiretroviral therapy	5 079	6 685	7 876	7 562
Specialist services	1 649	1 695	1 605	1 544
Other	169	206	256	171
HIV+ subjects on ART (n)	1 975	2 421	2 854	3 130
Mean age (years)	41.6	43.7	45.9	47.8
*Per capita cost* (€)				
Total	10 419	11 920	12 645	11 589
In-hospital	1 805	1 625	1 441	1 262
Antiretroviral therapy	6 796	8 393	9 342	8 612
Specialist services	1 649	1 695	1 605	1 544
Other	169	206	256	171
New HIV+ cases (n)	523	395	385	370
Mean age (years)	38.7	40.6	40.0	39.7
*Per capita cost* (€)				
Total	7 978	7 838	10 040	9 731
In-hospital	4 048	3 666	4 699	4 421
Antiretroviral therapy	2 522	2 554	3 589	3 496
Specialist services	1 362	1 608	1 705	1 760
Other	46	10	48	54

We also evaluated the association between, age, gender, period of follow-up, CD4^+^ cell count at baseline and chronic diseases with per capita cost using multivariate regression models (Table 5). The per capita cost was positively associated with age (+36€ every year of age), years of follow-up and CD4^+^ cell count at baseline (+2 452€ for subjects with CD4^+^ cell count <200 mm^3^ at baseline respect to subjects with CD4^+^ cell count ≥ 500 mm^3^) and the presence of chronic diseases. In particular, we observed an increase of 3 700€ for subjects with, compared to those without, at least one chronic disease. Kidney failure, psychiatric diseases and cancer were the most expensive comorbidities, with an increase of per capita cost of 13 665€, 8 172€ and 7 557€, respectively.

**Table 5:** Association of chronic diseases, age, gender, period of follow-up and CD4^+^ cell count with per capita cost of care for HIV^+^ subjects using multivariate regression models

	Per capita cost € (95 % CI)	*p*-value
Age (per year)	36.1 (24.5–47.8)	<0.001
Gender (male vs female)	−12.5 (−198.6–173.6)	0.895
Period of follow-up		
2003–2005	Ref	
2006–2008	1473.4 (1215.8–1731.0)	<0.001
2009–2011	2293.9 (2054.2–2533.6)	<0.001
2012–2014	1453.0 (1207.8–1698.2)	<0.001
CD4^+^ cell count		
≥ 500	Ref	
350–499	1272.0 (939.0–1605.1)	<0.001
200–349	1082.6 (864.7–1300.4)	<0.001
< 200	2452.3 (2238.7–2666.0)	<0.001
Prevalence of chronic diseases^a^		
Cardio-cerebrovascular diseases (yes vs no)	2087.8 (1736.1–2439.5)	<0.001
Liver diseases (yes vs no)	4388.8 (3786.0–4991.7)	<0.001
Dyslipidemia (yes vs no)	1003.4 (593.2–1413.5)	<0.001
Diabetes (yes vs no)	1701.4 (1125.0–2277.8)	<0.001
Cancer (yes vs no)	7557.6 (6737.0–8378.2)	<0.001
Gastrointestinal diseases (yes vs no)	3756.8 (2797.7–4715.8)	<0.001
Neuropathies (yes vs no)	2767.6 (2081.9–3453.3)	<0.001
Serious psychiatric diseases (yes vs no)	8172.2 (7056.0–9288.5)	<0.001
Chronic respiratory diseases (yes vs no)	4103.3 (3192.4–5014.1)	<0.001
Kidney failure (yes vs no)	13665.3 (11075.2–16255.5)	<0.001
At least one chronic disease (yes vs no)	3699.7 (3509.9–3889.5)	<0.001

^a^model adjusted for age, gender, period of follow-up, CD4 cell count. Abbreviation: *CI* confidence interval

## Discussion

This population-based study carried out in a Health Care District of North Italy is an extension of a previously published assessment of HIV-infection -related health care costs in 2003–2007 up to 2014 [10].

The prevalence of HIV infection increased from 220 to 307 per 100 000 person-years (+2.5 % annual mean change) while incidence decreased from 16 to 11 per 100 000 person-years (-4.5 % annual mean change) and mortality decreased from 25.6 to 13.4 per 1000 person-years (−8.7 % annual mean change). Concomitantly with aging of the cohort of HIV-infected patients, the prevalence of most chronic diseases increased across time and was higher than that of LHA general population. However, the observed to expected ratios (SPRs) in HIV^+^ subjects decreased over time for each chronic disease except dyslipidemia. Average per capita total cost of HIV infection increased from 2003 to 2014 (+25 %) mainly due to cost for ART (+49 %). Immunodeficiency level at the time of HIV diagnosis had an important weight on healthcare spending: the per capita cost for patients with CD4^+^cell count <200 mm^3^ was 2 106€ more than patients with CD4^+^cell count >500 mm^3^.

There is mounting evidence that an increase of use on HIV healthcare services leads to a reduction in both mortality and HIV transmission and therefore to reduction of HIV incidence [14–16]. Accordingly, we found an increase of HIV management costs and HIV prevalence and concurrently a decrease of incidence and mortality of HIV-infected patients over the period 2003–14. These findings appear to confirm that spending on public health for HIV management improves patients’ survival and prevents new HIV infections.

However, in spite of the increase of total costs for HIV infection care in the period (+25 % during the period), when ranking total costs for National Health Service of all chronic diseases in the Brescia LHA, HIV infection maintained the third rank in order of cost per patient since 2007 [10] to 2014 (present study).

A recent systematic review about economic impact of HIV infection in five European countries showed a high degree of inter-country variability, the HIV^+^ patients cost ranging from 6 399€ in Italy and 11 638€ in Spain to 25 339€ in UK and 32 109€ in Germany, ART being the main component of total HIV health cost [17].

In the same line, the increase of total cost for HIV in our study was entirely attributable to increase in ART cost, mainly due to the proportion of patients on ART increased +23 % over the period and because of introduction of new antiretroviral drugs approved from 2005, including new drugs within conventional classes as well as novel drug classes. These newer antiretroviral drugs are more effective in treatment of drug resistant HIV but, on the other hand, are also more expensive than those used previously, causing substantial incremental costs [5, 6].

The cost of ART for HIV^+^ incident cases was around half of that for all HIV^+^ patient across the period. The main reasons for this is that cheaper drugs are more commonly used in the first-line regimen than in the following ART lines [18]. Indeed, various authors [6, 19] have shown that the most powerful determinant of ART cost was the line of ART. In our cohort, more than 70 % of patients had used at least four lines of ART and 92 % of patients on ART had plasmatic HIV RNA <37cp/ml. (Data not shown in table). Furthermore, the complexity of HIV^+^ patients plays an important role in cost of ART including difficulty of achieving virological suppression in ART experienced patients, toxicities, comorbidities and drug-drug interaction thus contributing to the differences in ART cost among centers also inside the same country [6, 20].

In our study however, per capita costs for HIV^+^ patients increased from 2003 to 2011 and decreased moderately afterwards. This is probably due to the local public health policy. Indeed, in the context of present austerity policies, from 2011 the Lombardy Government has forced health services to increase efficiency of resources allocation and treatment appropriateness [Therapeutic and diagnostic path-PDT- [21]]. As a consequence, HIV- specialists are urged to the “economic efficacy principle” for choosing equally efficient but lower-cost alternative medication and generic drugs whenever available, according to PDT of the Lombardy Health Service [21].

The increase of life expectancy of HIV^+^ patients in the last decades due to efficacy of ART has determined patients’ aging with consequent increase of comorbidities and related health expenditure [9, 22]. HIV care involves taking ART and having regular check-ups by healthcare providers with opportunity for early diagnosis of comorbidities. Notwithstanding, HIV^+^ patients with comorbidities have poorer health outcomes than those without comorbidities [23]. The prevalence of chronic diseases was higher in HIV+ patients than in the LHA general population, in agreement with other studies showing that HIV+ patients have an early aging with a prevalence of multi-comorbidities approximately equivalent to that observed in the general population of 10–15 years older [24, 25]. The prevalence of many “non-AIDS” chronic diseases is inversely related to CD4^+^ cell count. This includes liver diseases [26], non-AIDS malignancies [27, 28] and renal diseases [29]. Whereas, other diseases, particularly cardio-cerebrovascular diseases do not seem to follow this pattern [30].

We observed a significant decreasing trend of the age and gender adjusted prevalence of at least one chronic disease adjusted for age and gender in HIV^+^ subjects over time (annual mean change − 0.7 %; p = 0.006). One possible explanation for this is that continuous treatment of HIV^+^ subjects in the period has determined an increase of CD4^+^ cell levels with consequent decreasing comorbidity, in agreement with the decrease of non-AIDS morbidity concurrently with CD4^+^ cell level increase in some HIV+ cohorts [2–4].

Although prevalence of all chronic diseases in our HIV^+^ patients was higher than the general population, the observed to expected ratio (SPR) for HIV^+^ subjects reduced in the period, especially for liver diseases. At present, HCV infection is the most common cause of chronic liver diseases in both HIV^+^ patients and the general population in Italy [31–33]. HIV/HCV coinfected subjects are less frequently treated for HCV than HCV-monoinfected ones due to higher difficulties to treat the formers due to immunodeficiency, drug-drug interaction, low tolerability, concomitant intravenous drug use or presence of psychiatric diseases. Furthermore, in Italy HIV^+^ subjects have a HIV reimbursement code allowing them to have whole health care totally free, including HCV management and cure. Therefore, HIV-HCV coinfected subjects do not ask for HCV reimbursement code, and, as a consequence, they are not detected by the LHA health database.

CD4^+^ cell count at baseline was inversely related with per capita cost and this cost was particularly high in HIV^+^ subjects at advanced immunodeficiency stage, in agreement with previous studies [6, 19, 34–36]. The Brescia LHA health status database is a large, population-based comprehensive and integrated information system which has the usual limits of currently available large databases of health data coming from various sources, leading to a certain degree of imprecision in estimating the frequency of chronic diseases and the cost of care of HIV^+^ subjects. Anyway, a validation study and various analyses have shown a fairly high quality of the database, especially as regards the most common chronic diseases including HIV, cardiovascular diseases, cancer and diabetes [37, 38].

As regards external validity, i.e. generalizability, the HIV patients living in the Brescia LHA are substantially similar to the national ones, in spite of a higher HIV incidence in Brescia than in the whole country (10.8/100000 vs 6.1/100 000 inhabitants). In fact, the HIV cases with first diagnosis in 2014 are similar to those described in the National reports in the same year as regards demographic characteristics (79 % vs 80 % males, mean age 38.8 vs 39 years, 23.8 % vs 27 % non-Italian citizens) [39]. Likewise, as regards clinical aspects, the proportion of HIV incident cases with CD4 level <200 cell / mm3 in our cohort was similar to the national one (33 % vs 35 %) [39].

Another possible limitation is that inflation has not been accounted for. However, the official mean inflation yearly rate in Italy in the period 2003–2104 has been 1.9 % (range: 0.2 % − 3.2 %) [40], hardly impacting on our data and results.

This study has some strengths, too. First, it is population-based allowing to estimate incidence, prevalence and mortality rates for all HIV^+^ subjects and all residents living in the area and therefore avoiding selection bias. Separate economic analyses could be performed for incident and prevalent HIV^+^ cases whereas most studies considered all HIV^+^ cases together. The relatively long observation time (12 years) allowed us to assess the temporal trend of the epidemiological pattern, burden of chronic diseases, and overall and specific costs of HIV^+^ subjects. Finally, all HIV^+^ patients have been cured in the unique Infectious Diseases Unit in the area, allowing the collection of clinical data and matching them with the LHA data base.

## Conclusions

Our findings suggest that spending on public health for HIV care can improve prognosis of HIV-infected patients, reduce transmission of HIV infection and reduce the global burden of chronic diseases, leading to a reduction of HIV global cost in the medium-long time.

## Additional file

Additional file 1: Supplementary Materials. Table S1 and Table S2. (DOC 81 kb)

## Abbreviations

ACC: Acces to continuity of care
ART: Antiretroviral therapy
BLHDB: Brescia local health agency data base
DRGs: Diagnosis related groups
LHA: Local health agency
NHS: National health service
SMR: Standardized mortality ratio
SPR: Standardized prevalence ratio
