# Supplementary material for: The burden of chronic diseases and cost-of-care in subjects with HIV infection in a Health District of Northern Italy over a 12-year period compared to that of the general population
Source: BMC Public Health. 2016 Nov 9;16:1146. doi: 10.1186/s12889-016-3804-4 (PMC5103392; doi:10.1186/s12889-016-3804-4)
Supplement: Supplementary file 1 — Supplementary Materials. Table S1 and Table S2. (DOC 81 kb) [file 12889_2016_3804_MOESM1_ESM.doc]

**Additional file 1: Table S1.** Methodology used to rank chronic co-morbid conditions (see methodological work by Lonati F., Scarcella C., Indelicato A. et al. Epidemiol Prev 2008; 32: 137-44).

| **Chronic diseases** | **Hospital Discharge Diagnoses**  **(DRG-ICD9CM**) | **Drug Prescription**  **(ATC/DDD**) | **Out-paient**  **care** | **Registry Residential care or Registry psychiatric facility**  **(SVM code)** | **Free access for care service** |
| --- | --- | --- | --- | --- | --- |
| **Chronic respiratory diseases** | 493*; 518.81; 491.2* | ATC=R03*  (DDD for the year ≥ 30%) |  | Respiratory disease diagnosed at admission (moderate or severe) | Asthmas  Chronic respiratory failure |
| **Cardio-Cerebrovascular diseases** (Hypertension, heart disease, cardiac insufficiency, cardiovasculopathies) | 394*, 395*, 396*, 397*, 401*, 402*, 403*, 404*, 405*, 414*, 424*, 426*, 427*, 429.4*, 745*,746*, V42.2; V43.3; V45.0*, 416*; 417*, 428.0; 428.1; 428.9, 416*, 417*, 433*, 434, 440*, 441.2*, 441.4*, 441.7*, 441.9*, 442*, 444*, 447.0, 447.1, 447.6, 452*, 453*; 459.1, 557.1, 747*, V43.4, 443.1 | C01, C02, C03, C07, C08, C09, BO1  (DDD for the year ≥ 70%) |  | Cardiovascular disease diagnosed at admission (moderate to severe) | Hypertension  Heart disease  Cardiovasculopathies |
| **Diabetes** | 250* or DRG 294 and 295 | A10A (DDD≥10%), or A10B (DDD≥30%) |  | Diabetes diagnosed at admission | Diabetes |
| **Dyslipidemia** | 272.0, 272.2, 272.4, 272.9 | C10 (DDD≥70%) |  | Dyslipidemia diagnosed at admission | Dyslipidemia |
| **Liver diseases** | 571.2, 571.5, 571.6, 070.32, 070.33, 070.54, 170.9, 571.4, 555*,556*, 577.1 DRG 202 |  |  | Liver disease diagnosed at admission (moderate or severe) | Cirrhosis/ Chronic hepathitis/ Ulcerative colitis and Crohn’s disease/ Chronic pancreatitis |
| **Gastrointestinal disease** | 272.0, 272.2, 272.4, 272.9 | A02B (DDD≥80%) |  | Esophago-gastro-duodenum disease diagnosed at admission |  |
| **HIV infection and AIDS** | 042*, V08 or DRG 488, 489 and 490 |  |  |  | HIV/AIDS |
| **Kidney failure** | 585, V56 or DRG 316 and 317 |  | Dialysis service | Chronic kidney failure diagnosed at admission (moderate or severe) | Chronic kidney failure |
| **Cancer** | 140*-208*; V10*, V58.1 | L01 | Oncology services chemo- and radiotherapy |  | Several kind of cancer |
| **Neuropathies** | 345*, 332*, 333.0, 333.1, 333.5, 331.5, 331.0, 340*, 341.0, 290.0, 290.1, 290.2, 290.4, 291.1, 294.0 |  |  | Neuropathies diagnosed at admission (moderate or severe) | Epilepsy/ Parkinson/ Alzheimer/ Multiple sclerosis/ Optic neuritis/ Dementia |
| **Severe psychiatric disorders** | Admission in hospital-, outpatient-, residential care for psychiatric disorders (division discharge code equal to 40), admission for MDC 19, DRG 424, 425, 426, 427, 428, 429, 430, 431, 432 |  |  | Admission in psychiatric health services or Psychiatric disorder diagnosed at admission (severe) | Psychiatric disorders |

**Legend to Appendix:** ICD-9-CM: International Classification of Disease 9^th^ Revision, Clinical Modification; DRGs: Diagnosis Related Groups; ATC: Anatomic and Therapeutic Chemical Classification; DDD: Daily Defined Doses.

**Additional file 1: Table S2. Prevalence and public health costs for 15 chronic diseases categories in the Brescia Local Health Agency (annual cost in 2014).**

| **Chronic diseases** | **Number of affected people** | **Prevalence per 1000 receiving care** | **age (mean)** | **Per capita cost (h)** | | | | | **Total health expenditure** |
| --- | --- | --- | --- | --- | --- | --- | --- | --- | --- |
|  |  |  |  | **Total** | **In-Hospital** | **Drugs** | **Diagnostic and specialistic services** | **Other*** |  |
| **Transplantation** | 1 815 | 1.5 | 54.6 | 20 080 | 10 477 | 5 684 | 3 902 | 17 | € 36 444 656 |
| **Kidney failure** | 6 376 | 5 3 | 71 3 | 12 425 | 4 885 | 1 571 | 4 997 | 972 | € 79 223 266 |
| **HIV infection** | 3 483 | 2 9 | 47 2 | 10 446 | 1 265 | 7 597 | 1 413 | 171 | € 36 383 871 |
| **Severe psychiatric disorders** | 11 754 | 9 8 | 61 6 | 10 113 | 2 524 | 589 | 446 | 6 554 | € 118 864 088 |
| **Neuropathies** | 22 365 | 18 7 | 67 6 | 6 799 | 2 559 | 1 226 | 616 | 2 397 | € 152 065 003 |
| **Cancer** | 52 455 | 43 9 | 65 2 | 5 591 | 2 732 | 1 346 | 1 298 | 215 | € 293 283 091 |
| **Gastrointestinal diseases** | 30 119 | 25 2 | 70 0 | 4 942 | 2 085 | 1 462 | 1 210 | 185 | € 148 844 454 |
| **Chronique respiratory diseases** | 34 493 | 28 9 | 57 4 | 4 555 | 2 432 | 1 035 | 593 | 495 | € 157 126 066 |
| **Liver diseases** | 17 238 | 14 4 | 57 3 | 3 836 | 1 580 | 1 185 | 729 | 342 | € 66 126 985 |
| **Diabetes** | 66 268 | 55 4 | 67 5 | 3 688 | 1 764 | 833 | 706 | 385 | € 244 420 439 |
| **Rare Diseases** | 10 578 | 8 9 | 39 0 | 3 564 | 879 | 1 970 | 655 | 60 | € 37 699 569 |
| **Dyslipidaemia** | 57 459 | 48 1 | 68 4 | 3 387 | 1 653 | 916 | 690 | 128 | € 194 600 073 |
| **Cardio- and cerebrovascular diseases** | 235 502 | 197 0 | 69 2 | 3 208 | 1 517 | 708 | 596 | 387 | € 755 528 096 |
| **Autoimmune diseases** | 12 554 | 10 5 | 53 7 | 2 768 | 962 | 1 081 | 601 | 123 | € 34 744 814 |
| **Endocrine diseases** | 31 184 | 26 1 | 58 6 | 2 604 | 1 152 | 610 | 599 | 243 | € 81 196 743 |
| **Total with chronic diseases** | 355 076 | 297 1 | 62 9 | 3 025 | 1 318 | 763 | 591 | 352 | € 1 074 080 400 |
| **Total without chronic diseases** | 840 088 | 702 9 | 34 1 | 335 | 153 | 39 | 138 | 4 | € 281 579 436 |
| **Total receiving care** | 1 195 164 | 1 000 | 42 7 | 1 134 | 500 | 254 | 273 | 108 | € 1 355 660 183 |
